# Supplementary material for: Acute Kidney Injury after Lung Transplantation: A Systematic Review and Meta-Analysis
Source: J Clin Med. 2019 Oct 17;8(10):1713. doi: 10.3390/jcm8101713 (PMC6833042; doi:10.3390/jcm8101713)

Search terms for systematic review.

Database: Ovid MEDLINE (1395 articles)

1. exp acute kidney injury/

2. acute kidney injury$.mp

3. exp acute renal failure/

4. acute renal failure$.mp.

5. exp renal insufficiency/

6. renal insufficiency$.mp.

7. exp dialysis/

8. dialysis$.mp.

9. hemodialysis$.mp.

10. renal replacement therapy$.mp.

11. hemofiltration$.mp.

12. hemodiafiltration$.mp.

13. 1 or 2 or 3 or 4 or 5 or 6 or 7 or 8 or 9 or 10 or 11 or 12

14. Lung.mp

15. Pulmonary.mp

16. 14 or 15

17. Transplant.mp

18. Transplantation.mp

19. 17 or 18

20. 16 and 19

21. 13 and 20

Database: EMBASE (414 articles)

“acute kidney failure” AND “lung transplantation”

Cochrane Database (0 article)

"lung transplantation" AND "acute kidney injury"

Figure 1. Forest plots of the included studies assessing the pooled OR of mortality at one year among patients after lung transplantation with AKI. AKI, acute kidney injury.

Study name	Odds ratio	Lower limit	Upper limit	Z-Value	p-Value	Relative weight
Rocha et al	4.330	2.083	9.002	3.925	0.000	13.41
Arnaoutakis et al	0.470	0.197	1.122	-1.700	0.089	12.21
Wehbe et al	2.920	1.764	4.832	4.169	0.000	15.31
Hennessy et al	7.010	3.292	14.928	5.049	0.000	13.20
Fidalgo et al	2.810	1.152	6.853	2.271	0.023	12.03
Nguyen et al	1.730	0.420	7.128	0.759	0.448	8.13
Bennett et al	6.200	2.738	14.040	4.375	0.000	12.66
Shashaty et al	3.640	1.681	7.883	3.277	0.001	13.05
	2.986	1.722	5.179	3.894	0.000	

(Statistics for each study)

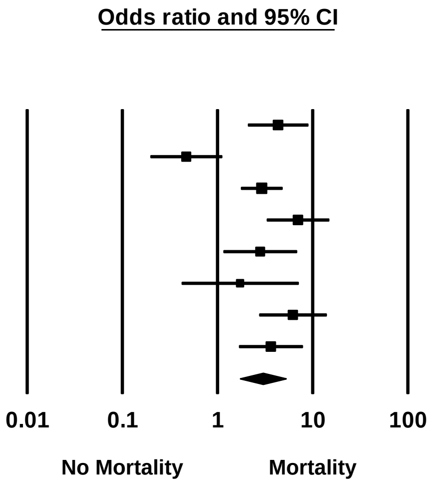

Figure S2: Forest plots of the included studies assessing the pooled OR of mortality at one year among patients after lung transplantation with severe AKI requiring RRT. AKI, acute kidney injury; RRT, renal replacement therapy.

Study name	Odds ratio	Lower limit	Upper limit	Z-Value	p-Value	Relative weight
Rocha et al	23.700	8.287	67.778	5.905	0.000	8.00
Arnaoutakis et al	4.970	1.542	16.020	2.685	0.007	6.73
George et al	7.890	6.802	9.152	27.278	0.000	35.11
Hennessy et al	43.040	9.478	195.453	4.873	0.000	4.34
Nguyen et al	1.200	0.317	4.550	0.268	0.789	5.41
Banga et al	7.230	6.200	8.431	25.239	0.000	34.94
Bennett et al	21.600	5.751	81.125	4.551	0.000	5.48
	8.321	5.953	11.632	12.399	0.000	

(Statistics for each study)

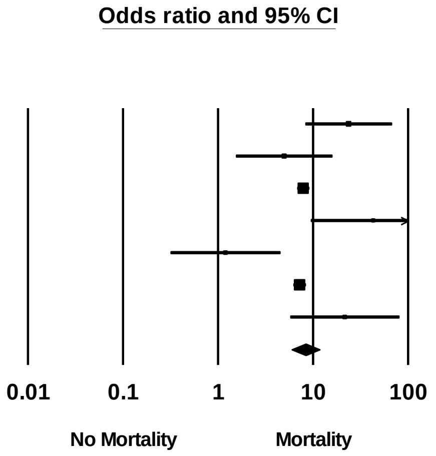

Figure S3: Forest plots of the included studies assessing the pooled OR of mortality at five years among patients after lung transplantation with AKI. AKI, acute kidney injury.

Study name	Odds ratio	Lower limit	Upper limit	Z-Value	p-Value	Relative weight
Rocha et al	1.440	0.901	2.302	1.523	0.128	36.25
Xue et al	1.480	1.039	2.108	2.172	0.030	63.75
	1.465	1.105	1.944	2.652	0.008	

(Statistics for each study)

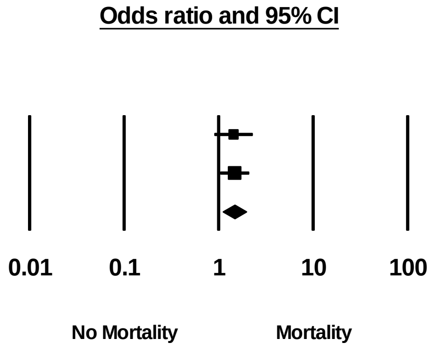

Figure S4: Forest plots of the included studies assessing the pooled OR of mortality at five years among patients after lung transplantation with severe AKI requiring RRT. AKI, acute kidney injury; RRT, renal replacement therapy.

Study name	Statistics for each study					Relative weight
	Odds ratio	Lower limit	Upper limit	Z-Value	p-Value	
Rocha et al	9.730	2.822	33.551	3.602	0.000	4.97
George et al	5.350	4.718	6.067	26.134	0.000	48.11
Banga et al	3.960	3.434	4.566	18.945	0.000	46.92
	4.786	3.581	6.396	10.583	0.000	

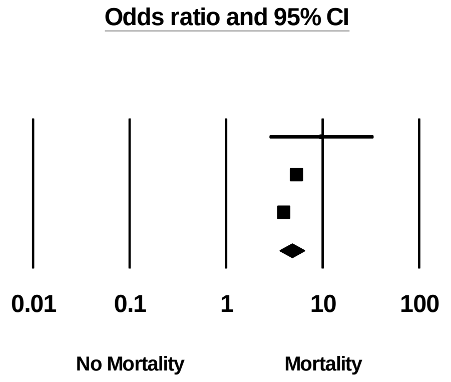

Figure S5: Funnel plot evaluating for publication bias evaluating mortality risk of AKI in patients after lung transplant with AKI. AKI, acute kidney injury.

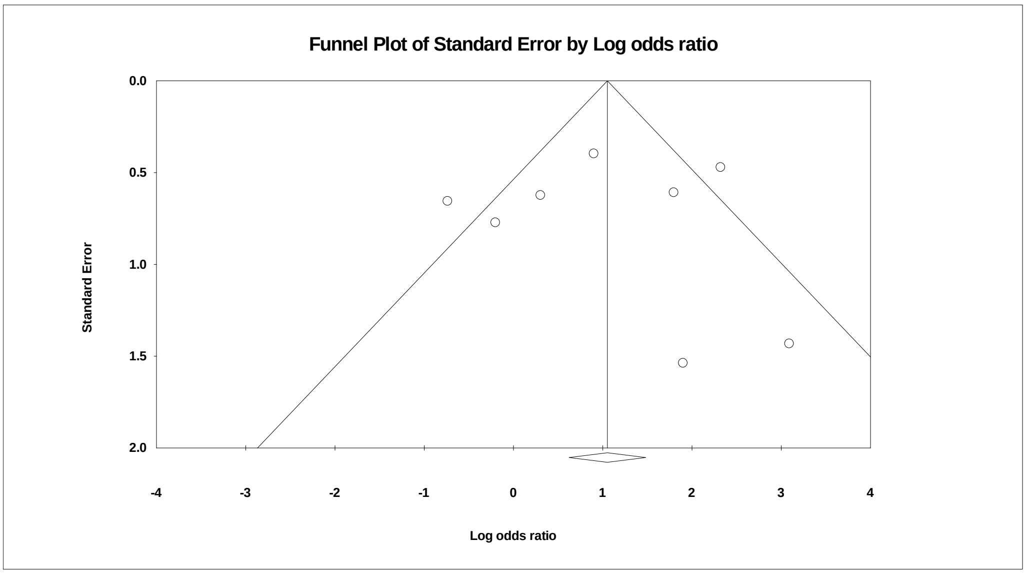

Figure S6: Funnel plot evaluating for publication bias evaluating mortality risk of AKI in patients after lung transplant with severe AKI requiring RRT. AKI, acute kidney injury; RRT, renal replacement therapy.

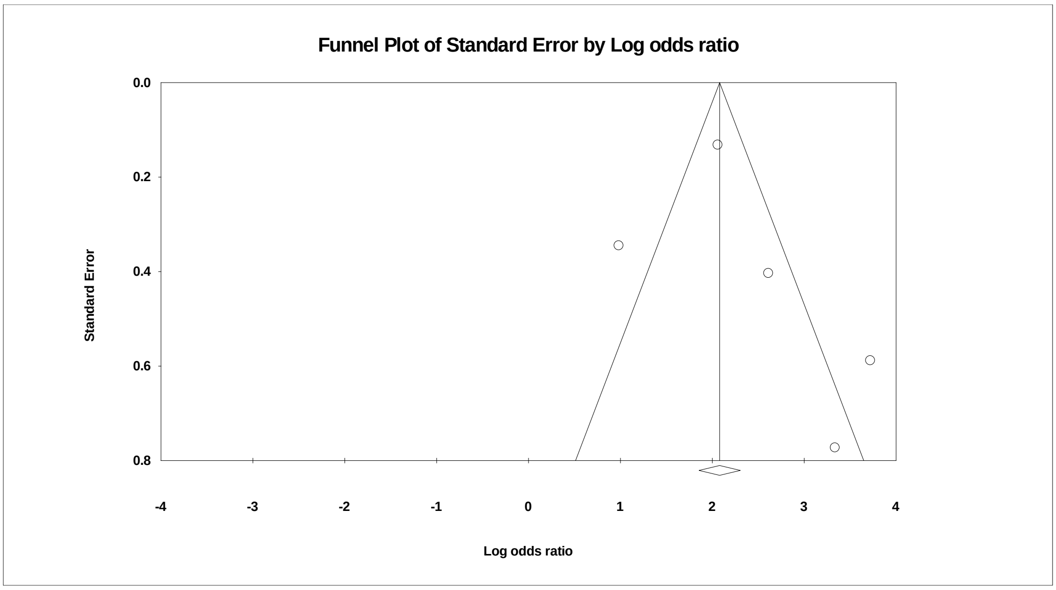

Supplement: Supplementary file 1 [file jcm-08-01713-s001.pdf]
